# Mitochondrial Biomarkers in Patients with ST-Elevation Myocardial Infarction and Their Potential Prognostic Implications: A Prospective Observational Study

**DOI:** 10.3390/jcm10020275

**Published:** 2021-01-13

**Authors:** Nicola Cosentino, Jeness Campodonico, Marco Moltrasio, Claudia Lucci, Valentina Milazzo, Mara Rubino, Monica De Metrio, Ivana Marana, Marco Grazi, Alice Bonomi, Fabrizio Veglia, Gianfranco Lauri, Antonio L. Bartorelli, Giancarlo Marenzi

**Affiliations:** 1 Centro Cardiologico Monzino IRCCS, 20138 Milan, Italy; jeness.campodonico@ccfm.it (J.C.); marco.moltrasio@ccfm.it (M.M.); claudia.lucci@ccfm.it (C.L.); valentina.milazzo@ccfm.it (V.M.); mara.rubino@ccfm.it (M.R.); monica.demetrio@ccfm.it (M.D.M.); ivana.marana@ccfm.it (I.M.); marco.grazi@ccfm.it (M.G.); alice.bonomi@cardiologicomonzino.it (A.B.); fabrizio.veglia@ccfm.it (F.V.); gianfranco.lauri@ccfm.it (G.L.); antonio.bartorelli@ccfm.it (A.L.B.); giancarlo.marenzi@ccfm.it (G.M.); 2 Department of Biomedical and Clinical Sciences “Luigi Sacco”, University of Milan, 20122 Milan, Italy

**Keywords:** ST-elevation myocardial infarction, cytochrome *c*, cell-free mitochondrial DNA, troponin I, prognosis

## Abstract

Background: Mitochondrial biomarkers have been investigated in different critical settings, including ST-elevation myocardial infarction (STEMI). Whether they provide prognostic information in STEMI, complementary to troponins, has not been fully elucidated. We prospectively explored the in-hospital and long-term prognostic implications of cytochrome *c* and cell-free mitochondrial DNA (mtDNA) in STEMI patients undergoing primary percutaneous coronary intervention. Methods: We measured cytochrome *c* and mtDNA at admission in 466 patients. Patients were grouped according to mitochondrial biomarkers detection: group 1 (−/−; no biomarker detected; *n* = 28); group 2 (−/+; only one biomarker detected; *n* = 283); group 3 (+/+; both biomarkers detected; *n* = 155). A composite of in-hospital mortality, cardiogenic shock, and acute pulmonary edema was the primary endpoint. Four-year all-cause mortality was the secondary endpoint. Results: Progressively lower left ventricular ejection fractions (52 ± 8%, 49 ± 8%, 47 ± 9%; *p* = 0.006) and higher troponin I peaks (54 ± 44, 73 ± 66, 106 ± 81 ng/mL; *p* = 0.001) were found across the groups. An increase in primary (4%, 14%, 19%; *p* = 0.03) and secondary (10%, 15%, 23%; *p* = 0.02) endpoint rate was observed going from group 1 to group 3. The adjusted odds ratio increment of the primary endpoint from one group to the next was 1.65 (95% CI 1.04–2.61; *p* = 0.03), while the adjusted hazard ratio increment of the secondary endpoint was 1.55 (95% CI 1.12–2.52; *p* = 0.03). The addition of study group allocation to admission troponin I reclassified 12% and 22% of patients for the primary and secondary endpoint, respectively. Conclusions: Detection of mitochondrial biomarkers is common in STEMI and seems to be associated with in-hospital and long-term outcome independently of troponin.

## 1. Introduction

Primary percutaneous coronary intervention (pPCI) is the most effective strategy for reducing infarct size and improving clinical outcomes in patients presenting with ST-segment elevation myocardial infarction (STEMI) [1]. Although reperfusion is essential for myocardial salvage, it can induce myocardial injury and cardiomyocyte death, thereby decreasing the beneficial effects of coronary revascularization [2].

The mitochondria are fundamental elements of cardiac function, as they supply the cell with essential biologic energy, through adenosine-three-phosphate (ATP) production [3]. Several experimental models have recognized that mitochondrial dysfunction is a critical factor in causing myocardial ischemic and reperfusion injury [4,5,6], directly contributing to reducing cardiac contractility. Thus, mitochondrial impairment in STEMI may increase patient vulnerability to ventricular dysfunction and death. On these bases, biomarkers that provide information on mitochondrial function in STEMI patients may be of clinical value to improve prognostic stratification and to find novel potential therapeutic targets [7].

Mitochondrial biomarkers, such as cytochrome *c* [8,9,10,11,12] and cell-free mitochondrial DNA (mtDNA) [13,14,15,16,17,18,19,20,21] have been studied in various clinical settings. Elevated circulating levels have been associated with poor prognosis, including mortality, after cardiac arrest and in other critical conditions [8,9,10,11,12,13,14,15,16,17,18,19,20,21]. Preliminary studies have suggested that detection of cytochrome *c* and mtDNA in the serum is linked with a worse outcome also in patients with acute myocardial infarction [11,12,19,20,21]. However, no study has prospectively evaluated the prognostic value of the two biomarkers, particularly when considered in combination, in terms of in-hospital and long-term outcomes in STEMI patients treated with pPCI. Specifically, it is unknown whether they share clinical and prognostic meanings with acknowledged markers of myocardial necrosis, in particular troponins [22,23], or if they are of additional predictive value.

Thus, in this study, we measured circulating cytochrome *c* and mtDNA at hospital admission in consecutive STEMI patients undergoing pPCI to assess whether they have any in-hospital and long-term prognostic value additional to that provided by troponin.

## 2. Methods

### 2.1. Study Population

This was a prospective, observational study. We included all consecutive STEMI patients undergoing pPCI at the Centro Cardiologico Monzino, University of Milan, Milan, Italy, between 1 June 2013 and 31 January 2016. Patients were included if they presented within 12 h (24 h for STEMI complicated by cardiogenic shock) from symptom onset (characteristic chest pain lasting for at least 30 min, not responsive to nitrates, with electrocardiographic ST-segment elevation in two or more contiguous leads, or left bundle-branch block). The only exclusion criterion was lack of informed consent. The study was approved by the Ethical Committee of our center (R726-CCM764), and written informed consent was obtained from all participants. This investigation conformed to the principles outlined in the Declaration of Helsinki.

### 2.2. Study Protocol

A 24-h on-call interventional team performed pPCI according to standard clinical practice. Standard guide catheters, guide wires, balloon catheters, and second-generation drug-eluting coronary stents were used via a radial or femoral approach. Peri-procedural pharmacology therapy and post-stenting antithrombotic treatment were given according to institutional protocols and guideline recommendations [24].

At hospital admission (before pPCI), peripheral venous blood was drawn to measure serum cytochrome *c*, mtDNA, and troponin I (TnI). Troponin I was also measured every 6 h, up to 24 h, after it reached the peak value. Study patients were stratified into three groups according to cytochrome *c* and/or mtDNA detection: group 1 (−/−; no mitochondrial biomarker detected); group 2 (−/+; only one of the two biomarkers detected; and group 3 (+/+; both biomarkers detected). Left ventricular ejection fraction (LVEF) was measured with echocardiography in all patients, before or soon after pPCI. After hospital discharge, all patients were followed up for 4 years.

### 2.3. Study Endpoints

The primary endpoint of the study was the composite of in-hospital mortality, cardiogenic shock, and acute pulmonary edema. We used this combined endpoint because cardiogenic shock and acute pulmonary edema are more closely associated with acute ventricular dysfunction and mortality in STEMI [24]. Cardiogenic shock was defined as persistent systolic arterial pressure ≤80 mmHg and evidence of vital organ hypoperfusion caused by severe left ventricular dysfunction, right ventricular infarction, or mechanical complications of infarction, and not due to hypovolemia, hemorrhage, bradyarrhythmias, or tachyarrhythmias. Acute pulmonary edema was defined as severe respiratory distress, tachypnea, and orthopnea, with rales over the lung fields and arterial oxygen saturation <90% on room air prior to oxygen administration. To avoid interference, each patient could only account for one event classification. Long-term all-cause mortality was the secondary endpoint.

### 2.4. Laboratory Assays

Circulating cytochrome *c* (ng/mL) was measured in the serum by ELISA, using a commercially available enzyme-linked immunosorbent assay (Quantikine, R&D System Inc., Minneapolis, MI, USA), as previously described [12]. The lowest detection limit was 0.05 ng/mL. Levels of mtDNA were measured in the serum (copies/μL), as reported by Nakahira et al. [13]. All cytochrome *c* and mtDNA measurements were performed at the end of the study. Thus, all physicians directly involved in data collection and patient management were unaware of the results of these biomarkers. Troponin I (ng/mL) was measured by a two-site immune-enzymatic (“sandwich”) chemiluminescent technique, using the UniCell DXI 800 (Beckman Coulter, Fullerton, CA, USA).

### 2.5. Statistical Analysis

Continuous variables are presented as mean ± SD, and compared using the *t*-test for independent samples. Variables not normally distributed are presented as median and interquartile ranges, and compared with the Wilcoxon rank-sum test. Categorical data were compared using the chi-square test or the Fisher exact test, as appropriate. Spearman correlation was used to detect possible correlations between cytochrome *c* and mtDNA.

Trends across the three study groups were assessed by one-way analysis of covariance (ANCOVA) and by Mantel–Haenszel chi-square, as appropriate. The association between the three groups and the primary endpoint was assessed by logistic regression analysis, and odds ratios (OR; for trend) were adjusted for admission and peak TnI values. Cox proportional hazard model was also used to assess hazard ratio (HR; for trend) for long-term mortality associated with the three groups. The HR was adjusted for major known predictors of long-term mortality in STEMI (epidemiological approach): age, gender, anterior STEMI, LVEF, admission serum creatinine, and Killip class [12]. Kaplan–Meier analysis was used to generate time-to-event curves for four-year mortality in the three groups. Log-rank test was used to compare strata.

We calculated the integrated discrimination index (IDI) to evaluate the improvement of the models’ ability to discriminate between high-risk and low-risk patients without using categories [25]. The IDI reflects the change in discrimination slope (i.e., difference between the mean estimated risk for cases and non-cases) of the model, with study groups added to admission TnI compared to the model with only TnI.

A *p* value <0.05 was considered statistically significant. All analyses were performed using SAS version 9.4 (SAS Institute, Cary, NC, USA).

## 3. Results

Four-hundred-sixty-six STEMI patients treated with pPCI were included in the study. At hospital admission, cytochrome *c* was detectable in the blood of 167 (36%) patients (median 0.76 (0.60–1.13) ng/mL), while mtDNA was measurable in 426 (91%) patients (median 539 (243–1285) copies/μL). In particular, in 28 (6%) patients, the two biomarkers were not detectable (group 1); in 283 (61%), only one of the two biomarkers was detectable (group 2); and in 155 (33%) both biomarkers were measurable (group 3). In the whole study population, no correlation was found between cytochrome *c* and mtDNA levels (*R* = −0.04; *p* = 0.38). The clinical characteristics and in-hospital outcomes of the three patient groups are shown in Table 1. Cardiovascular risk factors, prior cardiovascular events, STEMI location, time-to-reperfusion, and admission TnI were comparable among groups. However, group 3 patients showed higher heart rate, blood glucose, and serum creatinine levels at hospital admission, suggesting greater hemodynamic impairment.

**Figure 1:**
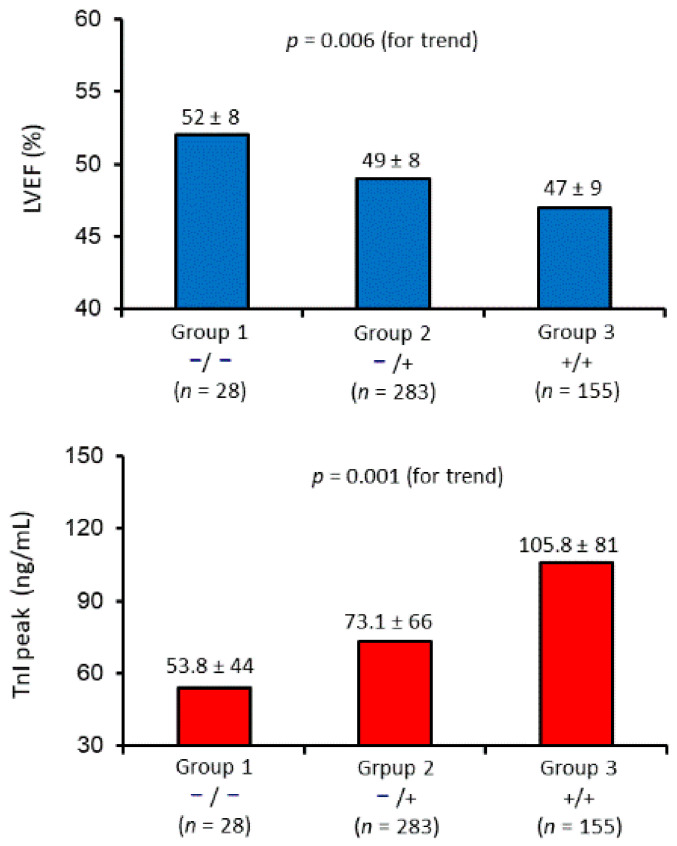
Left ventricular ejection fraction (LVEF; upper panel) and high-sensitivity troponin I (hs-TnI; lower panel) peak values stratified according to cytochrome *c* and/or mtDNA detection at hospital admission (group 1 (−/−; none mitochondrial biomarker detected) vs. group 2 (−/+; only one of the two biomarkers detected) vs. group 3 (+/+; both biomarkers detected)). *p* value for trend was obtained by one-way analysis of covariance (ANCOVA).

A progressive lower LVEF and a parallel increase of TnI peak values were found from group 1 to group 2 and 3 (Figure 1). Of note, the progressive LVEF reduction pattern was unchanged even after adjustment for TnI peak value. The geometric adjusted mean LVEF was 52 ± 10% in group 1, 49 ± 11% in group 2, and 47 ± 11% in group 3 (*p* = 0.04 for trend).

**Figure 2:**
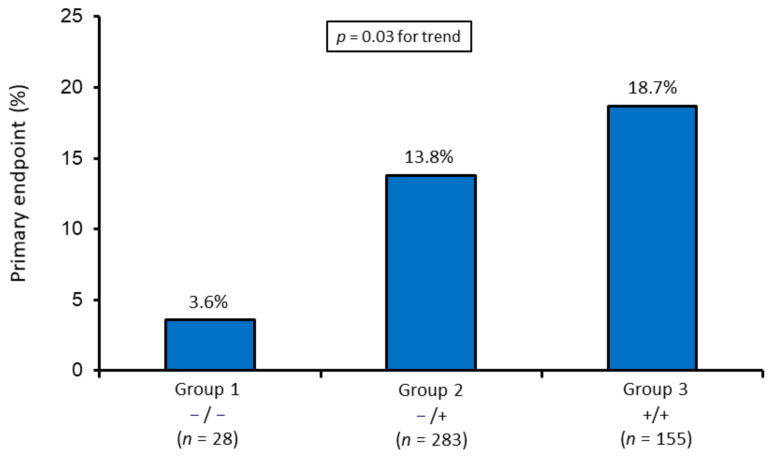
Rate of the primary endpoint (in-hospital mortality, acute pulmonary edema, and cardiogenic shock). Group 1 (−/−; no mitochondrial biomarker detected) vs. group 2 (−/+; only one of the two biomarkers detected) vs. group 3 (+/+; both biomarkers detected)). *p* value for trend was obtained by Mantel–Haenszel chi-square.

A significant progressive increase of the primary endpoint rate was also observed in the three groups (Figure 2). This was paralleled by an increasing risk of the primary endpoint, even after adjustment for admission and peak TnI values. The adjusted OR increment from one group to the next was 1.65 (95% CI 1.04–2.61; *p* = 0.03).

**Figure 3:**
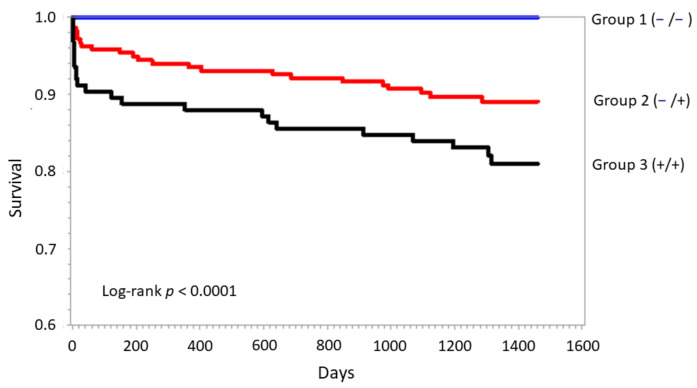
Kaplan–Meier curve analysis for long-term all-cause mortality stratified according to cytochrome *c* and/or mtDNA detection at hospital admission (group 1 (−/−; no mitochondrial biomarker detected) vs. group 2 (−/+; only one of the two biomarkers detected) vs. group 3 (+/+; both biomarkers detected)). *p* value was obtained by log-rank test.

In the overall population, the cumulative four-year mortality was 17% (*n* = 80). It was 10% in group 1, 15% in group 2, and 23% in group 3 (*p* = 0.02 for trend). The Kaplan–Meier curves of four-year mortality in the three groups are shown in Figure 3. The adjusted HR increment going from one group to the next was 1.31 (95% CI 1.04–1.89; *p* = 0.04).

At reclassification analysis, the addition of study group allocation according to mitochondrial biomarker detection to admission TnI allowed proper reclassification in 12% and 22% of patients for the primary and secondary endpoint, respectively (Table 2).

## 4. Discussion

The present study shows that cytochrome *c* and mtDNA were detectable at hospital admission in more than 30% and 90% of STEMI patients, respectively. Moreover, the two biomarkers seem to have prognostic implications complementary to those provided by admission TnI.

The incessant contractile function of the heart requires continuous energy supply that is sustained by a large amount of ATP provided by mitochondrial function [3]. Therefore, it is not surprising that in STEMI patients, alterations of mitochondrial energy balance due to myocardial ischemia-reperfusion injury may result in reduced contractile reserve and increased susceptibility to ventricular dysfunction and death [3,7,26].

Accumulating evidence suggests that mitochondrial biomarkers may be released into the circulation from injured cells, although the exact mechanism is still unclear [23]. In healthy subjects, cytochrome *c* is not detectable in peripheral blood, while circulating mtDNA may be found at very low concentrations [26]. On the other hand, high levels of cytochrome *c* and mtDNA were found in patients with several critical conditions, including stroke, sepsis, cardiac arrest, and massive pulmonary embolism [8,9,10,13,14,15,16,17,18,19,20,21,22,23,24,25,26,27,28]. Notably, their increment in these clinical settings has been consistently associated with a worse prognosis. Few preliminary studies reported an association between either cytochrome *c* or mtDNA and clinical outcome in acute myocardial infarction [11,12,19,20,21,29]. Thus, it may be hypothesized that mitochondrial impairment in STEMI, as reflected by the detection in blood of cytochrome *c* and/or mtDNA, could be associated with acute ventricular dysfunction and its major clinical consequences, namely acute pulmonary edema, cardiogenic shock, and short-term and long-term mortality. Moreover, it could be expected that this association is stronger in patients in whom both mitochondrial biomarkers are detectable.

To our knowledge, this is the first study exploring the combined prognostic impact of these mitochondrial biomarkers in a cohort of STEMI patients undergoing pPCI. This clinical scenario seems ideal for the purpose of our study because it is characterized by prolonged myocardial ischemia and abrupt myocardial reperfusion, both of which are well-known causes of mitochondrial injury [26].

We found that most (94%) STEMI patients have at least one circulating mitochondrial biomarker at hospital admission, while 30% of them had both biomarkers measurable. When patients were grouped according to detectable mitochondrial biomarkers (−/− vs. −/+ vs. +/+), a stepwise lower LVEF and higher TnI peak values were found. Notably, the significant LVEF reduction across the three groups was also confirmed after adjustment for TnI peak value, suggesting that the association between cytochrome *c*, mtDNA, and acute ventricular dysfunction was partially independent of myocardial necrosis. These findings were paralleled by a progressively higher rate of the primary in-hospital endpoint. In particular, the adjusted risk of the primary endpoint increased by 60% from group 1 (−/−) to group 2 (−/+), and by an additional 60% from group 2 to group 3 (+/+). A progressive trend in long-term mortality rate was also observed across study groups, with an almost 30% incremental risk even after adjustment for major prognostic predictors, including Killip class. Taken together, these findings suggest a greater ventricular dysfunction and a worse prognosis in STEMI patients when both mitochondrial biomarkers are detected.

The mechanisms underlying the association between release of mitochondrial biomarkers and in-hospital outcome cannot be inferred by our data. Although not all pathways involved in acute ventricular dysfunction during STEMI have been clarified completely, evidence indicates that myocardial stunning is implicated in addition to myocardial necrosis [27,28]. As mitochondria impairment during STEMI critically contributes to both myocardial necrosis and stunning [30], we can speculate that the lower LVEF, the higher TnI peak, and the worse clinical outcome paralleling the number of mitochondrial biomarkers detectable may reflect not only necrosis but also myocardial stunning. Indeed, release of cytochrome *c* to the cytosol and bloodstream was also demonstrated to occur without concomitant release of larger molecules, such as lactate dehydrogenase, which is considered a marker of cell necrosis [31]. In line with this hypothesis, group 3 patients had more severe hemodynamic impairment at hospital presentation, as reflected by higher heart rate, blood glucose, and serum creatinine, despite similar time-to-presentation and admission TnI values. Moreover, the progressive reduction in LVEF and incremental risk of the primary endpoint across the three groups were confirmed after adjustment for TnI peak value. This suggests that, along with myocardial necrosis, a sizeable number of cardiac cells may be still viable and only functionally impaired due to mitochondrial dysfunction. This leads to significant ATP depletion, ionic homeostasis imbalance, and therefore, depressed contractile force of cardiomyocytes [32]. Under these conditions, ATP will be mainly used to re-establish ion homeostasis rather than supporting contractile function [33]. How this theory can explain the long-term prognosis provided by the detection of the two mitochondrial biomarkers and their persistent ability to reclassify the risk of death at four years is less clear and needs further investigation. Of note, as shown in the Kaplan–Meier curves, the prognostic impact of mitochondrial biomarker detection was mainly driven by short-term mortality, reinforcing the potential role of myocardial stunning. In this regard, serial assessment of LVEF could help to clarify the link between the release of mitochondrial biomarkers and the transient or permanent cardiac dysfunction after STEMI and its influence on long-term outcome.

Although preliminary, our findings may pave the way to some relevant clinical implications. The combined measurement of cytochrome *c* and mtDNA at hospital admission might be useful to identify high-risk patients who could benefit from early implementation of therapies supporting ventricular contractility and reducing ventricular load [34]. Moreover, cardio-protective drugs preserving mitochondrial integrity and function, like cyclosporine A, metformin, and other manipulating mitochondrial agents, could have beneficial effects in patients with detectable mitochondrial biomarkers [35,36]. Indeed, several pharmacological agents capable of influencing selection of energy substrates in cardiac mitochondria have proven to exert a favorable impact in animal and clinical studies evaluating acute ischemia-reperfusion injury [32,35,36].

Several limitations of this study warrant mention. Firstly, we evaluated a small study population admitted to a single center. Secondly, because this was an observational study, a cause-effect relationship between detection of mitochondrial biomarkers and study endpoints cannot be established. Thus, our study can be considered as hypothesis generating only. Thirdly, how cytochrome *c* and mtDNA reach the bloodstream is not well understood and cannot be inferred from our data. To this regard, it should be highlighted that mitochondrial dysfunction is accompanied by autophagosomes induction during myocardial ischemia-reperfusion injury, which is important in maintaining mitochondrial function and might be involved in mitochondrial biomarker release [37]. Moreover, the biological meaning of these two molecules in the clinical setting of STEMI remains undefined. However, lack of correlation between them seems to suggest either a different biological meaning or different release kinetics. Finally, we did not serially evaluate cytochrome *c* and mtDNA levels in our patients. As all of them underwent pPCI, we cannot exclude that, if the two biomarkers had been measured not only at hospital admission but also after the procedure, their prognostic value could have been increased by incorporating mitochondrial damage associated with pPCI-related reperfusion injury.

In conclusion, detection of mitochondrial biomarkers is common among STEMI patients. Our results suggest that cytochrome *c* and mtDNA may be associated with acute ventricular dysfunction and with short-term and long-term outcomes with a prognostic role that seems to be complementary to that of troponin. Further studies are needed to investigate which are the mechanisms underlying serum increase in mitochondrial biomarkers in STEMI patients and whether their detection can be used to guide targeted therapies.

## Figures and Tables

**Table 1:** Clinical characteristics and in-hospital outcomes of study patients grouped according to cytochrome *c* and/or cell-free mitochondrial DNA (mtDNA) detection.

Variable	−/− (*n* = 28)	−/+ (*n* = 283)	+/+ (*n* = 155)	*p* Value *
Age (years)	65 ± 12	66 ± 12	66 ± 12	0.57
Male sex, *n* (%)	22 (79%)	208 (73%)	115 (74%)	0.77
Body weight (kg)	79 ± 18	76 ± 16	76 ± 15	0.47
Diabetes mellitus, *n* (%)	6 (21%)	64 (23%)	35 (23%)	0.41
Hypertension, *n* (%)	10 (36%)	160 (57%)	92 (50%)	0.09
Dyslipidaemia, *n* (%)	10 (36%)	117 (42%)	66 (43%)	0.60
Smokers, *n* (%)	13 (46%)	157 (56%)	70 (45%)	0.12
Prior MI, *n* (%)	4 (14%)	47 (17%)	23 (15%)	0.79
Prior CABG, *n* (%)	1 (4%)	17 (6%)	11 (7%)	0.58
Prior PCI, *n* (%)	1 (4%)	54 (19%)	24 (15%)	0.83
Anterior STEMI, *n* (%)	11 (39%)	110 (39%)	67 (43%)	0.18
Time-to-treatment (hours)	5.0 (2–7)	3.0 (2–5)	3.0 (2–6)	0.59
Admission heart rate (bpm)	70 ± 15	76 ± 16	81 ± 22	0.03
Admission SAP (mmHg)	130 ± 12	134 ± 25	135 ± 23	0.76
Laboratory values at hospital admission				
Troponin I (ng/mL)	9.8 ± 7.0	8.7 ± 7.3	9.1 ± 7.2	0.49
Blood glucose (mg/dL)	152 ± 63	158 ± 60	180 ± 8	0.006
Serum creatinine (mg/dL)	0.99 ± 0.28	0.99 ± 0.33	1.09 ± 0.55	0.03
Hemoglobin (g/dL)	14.2 ± 1.5	14.0 ± 1.7	13.9 ± 2.0	0.41
hs-CRP (ng/mL)	14.2 ± 20.8	19.9 ± 38.6	26.6 ± 49.5	0.11
In-hospital outcomes, *n* (%)				
Death, *n* (%)	0 (0%)	7 (2%)	11 (7%)	0.01
Cardiogenic shock, *n* (%)	0 (0%)	21 (7%)	17 (11%)	0.05
Acute pulmonary edema, *n* (%)	1 (4%)	29 (10%)	26 (17%)	0.01
Invasive MV, *n* (%)	1 (4%)	15 (5%)	22 (14%)	0.001
AKI, *n* (%)	1 (4%)	16 (6%)	14 (9%)	0.15
Atrial fibrillation, *n* (%)	1 (4%)	27 (10%)	20 (13%)	0.14
High-degree A-V block, *n* (%)	2 (7%)	14 (5%)	7 (5%)	0.63
VT/VF, *n* (%)	5 (18%)	27 (10%)	16 (10%)	0.49
Blood transfusion, *n* (%)	0 (0%)	9 (3%)	3 (2%)	0.89

Notes: AKI = acute kidney injury (defined as serum creatinine increase ≥0.5 mg/dL); A-V = atrio-ventricular; CABG = coronary artery bypass graft surgery; hs-CRP = high-sensitivity C-reactive protein; MI = myocardial infarction; MV = mechanical ventilation; PCI = percutaneous coronary intervention; SAP = systolic arterial pressure; STEMI = ST-elevation myocardial infarction; VT/VF = ventricular tachycardia/ventricular fibrillation. * *p* for trend.

**Table 2:** Reclassification statistics comparison in the prediction of primary and secondary endpoints of study groups added to admission troponin I.

Primary Endpoint			
Model	IDI	(95% CI)	*p*
Admission troponin I plus study groups vs. admission troponin I	12%	2–26	0.03
Secondary endpoint			
Model	IDI	(95% CI)	*p*
Admission troponin I plus study groups vs. admission troponin I	22%	4–40	0.002

Notes: CI = confidence interval; IDI = integrated discrimination index.

## Data Availability

The data presented in this study are available on request from the corresponding author.
